# Targeting Metabolic Modulation and Mitochondrial Dysfunction in the Treatment of Heart Failure

**DOI:** 10.3390/diseases5020014

**Published:** 2017-05-10

**Authors:** Abbey Steggall, Ify R. Mordi, Chim C. Lang

**Affiliations:** 1 School of Medicine, Flinders University, Adelaide 5042, Australia; abbey.steggall@sa.gov.au; 2 Division of Molecular and Clinical Medicine, Mailbox 2, Ninewells Hospital and Medical School, University of Dundee, Dundee DD1 9SY, UK; i.mordi@dundee.ac.uk

**Keywords:** heart failure, metabolic, mitochondria, trimetazidine, ranolazine, perhexiline, elamipretide

## Abstract

Despite significant improvements in morbidity and mortality with current evidence-based pharmaceutical-based treatment of heart failure (HF) over the previous decades, the burden of HF remains high. An alternative approach is currently being developed, which targets myocardial energy efficiency and the dysfunction of the cardiac mitochondria. Emerging evidence suggests that the insufficient availability of ATP to the failing myocardium can be attributed to abnormalities in the myocardial utilisation of its substrates rather than an overall lack of substrate availability. Therefore, the development of potential metabolic therapeutics has commenced including trimetazidine, ranolazine and perhexiline, as well as specific mitochondrial-targeting pharmaceuticals, such as elamipretide. Large randomised controlled trials are required to confirm the role of metabolic-modulating drugs in the treatment of heart failure, but early studies have been promising in their possible efficacy for the management of heart failure in the future.

## 1. Introduction

Despite advances in modern therapeutics and a significant reduction in mortality and morbidity, the burden of heart failure (HF) remains significant [1]. It is estimated that there are 23 million people with heart failure worldwide, with a lifetime risk for both men and women for developing heart failure of one in five [1,2]. Heart failure causes around 5% of all adult hospital admissions [3], and in 2013, over 20% of deaths in the United States were determined to be attributed to heart failure [4]. HF mortality remains high despite the establishment of renin-angiotensin-aldosterone blockade and beta-blockade as mainstays of therapy [4]. Thus, emphasis on the development of alternative pharmacological strategies is required [2]. Traditionally, the modification of hemodynamic and neurohormonal alterations that occur in the failing heart have been the focus of treatment resulting in the use and development of drugs aimed at improving hemodynamics, such as inotropes, beta-blockers and angiotensin-converting enzyme (ACE) inhibitors [2,5]. However, with the failure of such therapies to improve heart failure statistics, recent efforts have been directed instead towards utilisation of improved knowledge of heart failure pathophysiology. This has led to recent interest in the development of therapeutics aimed at enhancing myocardial energy efficiency via preferential metabolism of glucose as a substrate, rather than fatty acids, in order to increase adenosine triphosphate (ATP) production per mole of oxygen consumed [2,6,7,8]. Furthermore, with increasing evidence that the dysfunction of cardiac mitochondria is pivotal in heart failure, the most recent medication developments have aimed to improve mitochondrial functioning to maximise energy production [9,10,11]. This paper will seek to define the metabolic and mitochondrial changes that occur in heart failure and explore the recent advances in therapeutic options that target this metabolism.

## 2. Metabolism in Heart Failure

The energy requirements of the myocardium are high and must be met in order to sustain cardiac contraction [12]. The healthy human heart hydrolyses greater than 6 kg of ATP per day in support of its normal functioning and workload [13]. Further, the capacity of myocardial ATP storage is comparatively low [12] resulting in a high dependence on the ability of the heart to function to capacity in relation to energy production. Aerobic cardiac ATP production is physiologically produced via mitochondrial oxidation of carbohydrates and fatty acids [14]. Further ATP can be sourced via alternate substrates, such as ketone bodies, in order to ensure the high energy requirements are met, referred to as a high degree of metabolic flexibility [12]. In normal functioning, the majority (around 70%) of cardiac ATP production is via fatty acid metabolism [3].

In patients with heart failure, the myocardium, despite being a metabolic omnivore in being able to utilise various substrates, is unable to produce sufficient ATP to meet metabolic demands. Heart failure is considered a condition of metabolic impairment due to the reduction in ATP production resulting in energetic starvation [8]. Often insufficient energy supply to the myocardium appears to not be as a result of a lack of substrate availability, but rather due to an abnormality in the myocardial utilisation of these substrates [13]. The impairment in energy production is exacerbated by increasing metabolic demands of the heart due to the excessive activation of the sympathetic nervous system known to occur in patients with heart failure [13]. The modifications to metabolism and their mechanism are complex, not fully understood and do differ among heart failure of different aetiologies, though there is clear evidence that there are severe metabolic alterations in any failing heart [6].

The initial response to the failing myocardium is a downregulation of fatty acid metabolism with a relative preservation or increase in glucose uptake [3]. There is a preferential glucose metabolism due to a downregulation of pyruvate dehydrogenase (PDH) isoforms, which normally exert an inhibitory effect on glucose oxidation [13]. Concurrently, there is a reduced expression of medium chain acyl-CoA dehydrogenase of greater than 40% compared to normal [8]. Acyl-CoA is involved in the process of fatty acid beta-oxidation, and with its downregulation, there is a net reduction in fatty acid metabolism [13]. Fatty acid oxidation is also regulated, and reduced in heart failure, by peroxisome proliferator-activated receptor (PPAR) nuclear receptors, which are important transcriptional regulators of fatty acid oxidation [6].

As the pathological state of heart failure progresses, with the establishment of a compensatory hyperadrenergic state, this initial response ultimately results in an elevated level of free fatty acids in the serum [13]. An excess of circulating fatty acids, greater than the heart’s oxidative capacity, results in the storage of free fatty acids as intramyocardial triglycerides. Such storage of triglycerides is associated with both lipotoxicity and worsening of the established heart failure [15]. The excess of circulating free fatty acids can also lead to the onset of insulin resistance [13]. Reduced insulin-dependent glucose uptake follows cardiac insulin resistance [8], and an impairment of glucose metabolism becomes evident as systolic dysfunction occurs in heart failure [6]. Further mechanisms of the changes in carbohydrate metabolism in heart failure are not fully understood, and contradictory evidence regarding cardiac glucose use exists [6]; though it is thought that the impaired oxidation of glucose may be attributed to by any combination of ‘mitochondrial dysfunction, reduced expression of genes involved in glycolysis and glucose oxidation or decreased abundance of the pyruvate dehydrogenase complex’ [6], involved in glucose oxidation.

Following insulin resistance and reduced glucose metabolism, the myocardium becomes increasingly reliant on the metabolism of free fatty acids, and upregulation of this metabolism occurs [15]. In comparison to glucose utilisation, free fatty acid oxidation requires a greater oxygen requirement with decreased mechanical efficiency and ultimately a reduced ATP yield [13]. Thus, fatty acid oxidation is considered to have an inferior energy efficiency, and following the shift toward dependence on fatty acid metabolism, cardiac oxygen consumption increases by 30–50% [13].

### Metabolism in Diabetic Cardiomyopathy

Diabetic cardiomyopathy, cardiac disease independent of vascular complications in diabetes, is a heart failure syndrome worth considering due to its pathophysiology association with metabolic abnormalities [16]. Early in diabetic cardiomyopathy, there is observable left ventricular hypertrophy and myocardial remodelling, which progresses to cause abnormal left ventricular filling and diastolic dysfunction followed by eventual systolic dysfunction [16]. Recent evidence suggests that alterations in cardiac metabolism that occur in diabetes mellitus contribute towards this contractile dysfunction [17], particularly due to the insulin resistance detected in the diabetic heart in numerous studies [18]. Similar to other forms of heart failure, the major metabolic abnormalities occurring in diabetic cardiomyopathy are elevations in fatty acid oxidation in the context of reduced glucose uptake and metabolism [16].

Diabetes mellitus results in alterations in carbohydrate metabolism, including impaired glucose uptake and reduced glycolysis and pyruvate oxidation [17]. A reduction in GLUT4 protein, the major cardiac glucose transporter, due to reduced transcription in diabetes [18], and impaired insulin signaling in the diabetic heart result in the observable reduction in glucose uptake [17]. Additionally, there is the promotion of reactive oxygen and nitrogen species by hyperglycemia, which leads to myocardial apoptosis [17].

In contrast to glucose, fatty acid uptake across the plasma membrane is insulin independent, allowing an augmentation of the delivery of circulating fatty acids to the myocytes for oxidation in diabetes [17]. The fatty acid translocase/CD36, upregulated in the diabetic heart, is thought to be important in the etiology of cardiac hypertrophy and diabetic cardiomyopathy [18]. There is also an increased availability of fatty acids, within the circulation, given the enhanced lipolysis in adipose tissue and higher lipoprotein synthesis in the liver in a diabetic patient [17]. Despite the advantage of increased lipolysis in the setting of reduced glucose oxidation to ensure adequate ATP production, eventually the fatty acid supply supersedes the oxidative capacity of the heart. Inevitably, excess fatty acids are stored as triglycerides causing lipotoxicity, as discussed above, contributing to the initiation and progression of cardiomyopathy [17]. Furthermore, as in heart disease, the oxidation of fat opposed to glucose requires greater oxygen consumption, reducing cardiac efficiency and placing further strain on the heart, potentiating disease progression [17].

A further factor influencing the development of heart disease in diabetics is the effect of the forkhead box-containing protein of the subfamily 0 (Fox0). Fox0 proteins have recently been identified as important targets of insulin and other growth factors, as they act in the myocardium [19]. Furthermore, Fox0 transcription factors have been identified to regulate cardiac growth, insulin signaling and glucose metabolism in the heart [19]. Animal studies have suggested that Fox0 may be persistently and pathologically activated in diabetes [20]. Excess activation of Fox0 is thought to commence when the cardiac substrate use changes, outlined above, occur and with cardiomyocyte insulin resistance [20]. The activation of Fox0 is considered likely to be significant in the metabolic changes that underlie diabetic cardiomyopathy, but further studies to develop this prediction are required [19].

## 3. Metabolic Modulating Therapeutics

This improved understanding of the role of metabolism in the pathophysiology of heart failure has prompted further research into potential metabolic therapies. The current European Society of Cardiology (ESC) guidelines outline that the major aims of medical management of heart failure are the alleviation of signs and symptoms, reduction in hospitalisations and decreased mortality [21]. Further, improved quality of life and increased functional capacity are also emphasised as important objectives of treatment, but are recognised as difficult to measure, limiting their use in clinical trials [21]. A number of upcoming agents targeting metabolic modulation will be reviewed to determine effectiveness regarding their ability to meet these objectives.

### 3.1. Trimetazidine

One metabolic modulator, which is already widely used in the treatment of stable angina pectoris and has a relatively significant amount of evidence for use in the treatment of heart failure, is trimetazidine [22]. Trimetazidine is administered orally in either immediate release 20-mg tablets, three times daily, or via twice daily dosing of 35-mg modified-release tablets [23]. Trimetazidine is an anti-ischemic agent, and the key known mechanism of action is via inhibition of the long-chain mitochondrial 3-ketoacyl coenzyme A thiolase enzyme, resulting in the reduction of myocardial fatty acid uptake and oxidation [23]. Partial inhibition of fatty acid oxidation enhances glucose oxidation, a more efficient means of ATP production as discussed earlier [24]. Another potentially beneficial mechanism of action is trimetazidine’s direct inhibition of cardiac fibrosis via reduction of collagen accumulation and reduced connective tissue growth factor (CTGF) expression [25].

There is currently a lack of properly designed and controlled large clinical trials evaluating the use of trimetazidine in heart failure, but a number of smaller studies and meta-analyses have been performed with promising results [25], as summarised in Table 1. Further, trimetazidine has been proven to have a high tolerability with an absence of adverse hemodynamic affects throughout its use in acute coronary syndrome, suggesting that despite the relative absence of high quality evidence, it could potentially be safely implemented in heart failure treatment in patients with angina based on the current evidence alone [26]. Trimetazidine has been shown to have no effect on resting blood pressure, while lowering resting heart rate by an average of just 2.6 beats per minute [22]. Therefore, trimetazidine can be effective and safe in complementing standard therapies that do target these hemodynamic parameters [23]. Moreover, trimetazidine has been shown to be increasingly effective, in improving cardiac function and reducing symptoms of heart failure, when combined with metoprolol, a class a beta 1–2 receptor blocker, than when treated with trimetazidine alone [27].

Trimetazidine has consistently been shown to be effective in improving the left ventricular ejection fraction (LVEF), reducing the New York Heart Association (NYHA) classification, decreasing the rate of hospitalisation and reducing the recorded brain natriuretic peptide (BNP) in patients with heart failure [22,28,30,32]. A number of studies has also determined that trimetazidine is effective in reducing all-cause mortality for patients with heart failure [29,31,32]. Zhang et al. [22] and Zhou and Chen [30] contraindicated this result and concluded that there was no significant difference in all-cause mortality. However, both studies considered that, given that cardiac cause of mortality was significantly reduced, there remained a therapeutic benefit of treatment with trimetazidine. There is further contradictory evidence regarding the statistical benefit of trimetazidine on exercise tolerance in heart failure. An increased exercise tolerance time of at least 30 s with treatment with trimetazidine compared to placebo has been reported [22,32]. Zhou and Chen [30] concluded that the difference in exercise tolerance was not significantly different to that of conventional therapy, though did acknowledge the limitation of the methodological quality of the included studies used in their meta-analysis.

### 3.2. Ranolazine

Ranolazine is a metabolic modulator, similar structurally to trimetazidine [7]. Ranolazine is a partial fatty acid beta-oxidation inhibitor, when high serum concentrations are achieved, with reciprocal increases in glucose oxidation and PDH activity [12]. However, the drug’s main mechanism of action appears to be the inhibition of the late inward sodium channels [13]. The physiological inactivation of this channel is disrupted in the failing myocyte leading to sodium-triggered calcium overload, causing contractile and electrophysiological disturbances [13], which can increase left ventricular diastolic tension and myocardial oxygen consumption [33]. Thus, in targeting the late sodium channel, ranolazine reduces calcium overload in the myocyte, which hypothetically improves diastolic tension and relaxation [34]. This action of ranolazine on the late inward sodium channels requires lower doses than those required to provide the drug’s cardioprotective effect [12].

Ranolazine is currently approved for use in chronic angina, and there is some evidence suggesting its clinical relevance in the treatment of heart failure [35], summarised in Table 2. The first clinical study that specifically sought to determine the potential beneficial effects of ranolazine in heart failure was in patients with preserved ejection fraction (Ranolazine for the treatment of Diastolic Heart Failure (RALI-DHF) Proof-of-Concept Study) [34]. Maier et al. hypothesised that ranolazine would improve diastolic dysfunction given its action in promoting calcium extrusion and following the success of animal studies [34]. The study did demonstrate a reduction in left ventricular end-diastolic pressure with ranolazine treatment compared to placebo, but could not determine if the small change in pressure, 2 mmHg, would have any clinical relevance [34]. Maier et al. also demonstrated an acute reduction of systolic function following ranolazine administration, but it was concluded that this finding did not preclude potential positive long-term effects [34]. Despite the small sample size of the RALI-DHF study, the authors determined that ranolazine could provide improvement in the measure of hemodynamics, but that there was no evidence of improvement in relaxation parameters [34].

More recently, Murray and Colombo investigated the benefit of ranolazine in both systolic and diastolic dysfunction and found an improvement in the left ventricular ejection fraction in both classes following ranolazine treatment [36]. Other benefits appeared to be a reduction in cardiovascular events, fewer deaths and reduced hospitalisations. However, the study design was non-randomised and underpowered, so only a potential benefit of ranolazine exists requiring further investigation. Further evidence may be provided imminently as a further randomised control trial is currently underway and has commenced enrolling patients. This trial, The Effects of Ranolazine on Exercise Capacity in Patients with Heart Failure with Preserved Ejection Fraction (RAZE trial), is aimed at determining the effect of ranolazine on exercise capacity in patients with heart failure with preserved ejection fraction [37].

Despite the limited availability of clinical trial data supporting the use of ranolazine, several experimental investigations indicate a benefit of ranolazine in the treatment of heart failure [38], summarised in Table 3. Experiments on right ventricular muscle strips from end-stage human failing hearts showed a clear and significant reduction in diastolic tension [39]. Canine studies have revealed promising uses of ranolazine; diastolic benefits of restoration of myocyte relaxation, reduction in resting tension and reduction in left ventricular end diastolic pressure have been observed [40,41]. Further, Rastogi et al. revealed improved ejection fraction and reduced end diastolic wall stress following treatment with ranolazine with exaggeration of this benefit when ranolazine treatment was combined with metoprolol or enalapril [42]. This evidence provides incentive for further research that could demonstrate a clinical benefit of ranolazine in heart failure in the future.

### 3.3. Perhexiline

Perhexiline is another metabolically-acting drug that was originally developed as an anti-anginal medication [43]. This drug was initially utilised in the 1970s; but the underlying mechanism was not understood, and its use substantially declined due to identification of serious adverse effects, including hepatotoxicity and neurotoxicity [7]. More recently, with improved understanding of the drug’s pharmacokinetics, the toxicity has been found to be preventable with individualised dosing and dosage titration to steady state levels between 150 and 600 mg/mL [7]. Perhexiline is now considered a relatively safe treatment for cardiac conditions, but remains difficult to use given the ongoing need to individualise dosing and ongoing monitoring of plasma levels [44].

Perhexiline was first demonstrated to act in reducing fatty-acid oxidation in a 1995 experiment monitoring the effect of perhexiline on rat hearts [45]. A reduction of fatty acid utilisation of 35% was recorded with a concurrent increase in cardiac output of 80 mL/min/g (*p* < 0.05). This understanding was extended by an additional rat study that identified perhexiline as an inhibitor of the enzyme carnitine palmitoltransferase-1 (CPT-1), which was known to control access of long chain fatty acids to the mitochondrial site of beta-oxidation [46]. Despite this improved understanding, it remains likely that further pharmacological mechanisms remain undiscovered, and an additional four mechanisms of the drug have since been reported with limited awareness of their influence on cardiovascular function, limiting the clinical application of perhexiline [47].

The potential benefit of perhexiline in heart failure was observed in a later experiment that recorded an improvement in rat heart diastolic function during ischemia [48]. However, this benefit was not retained during reperfusion, and perhexiline had no impact on systolic function [48]. Perhexiline is not yet available for use in heart failure, though a few small placebo-controlled clinical studies, summarised in Table 4, suggest it may be effective in the future, and it is currently safely used in treating chronic stable angina in Australia and parts of Asia [49].

Lee et al. conducted an initial clinical trial to determine the efficacy of perhexiline in heart failure, particularly its effect on the peak exercise oxygen consumption (VO_2_ max) [53]. Despite the small sample size and short follow-up period of eight weeks, a clear improvement in peak oxygen consumption was found following perhexiline treatment compared to no change in patients treated with a placebo [53]. This improvement was larger than that which has been observed to occur via treatment with ACE inhibitors and occurred in addition to optimal medical treatment [53]. Additional findings included evidence that perhexiline improved left ejection fraction, symptoms, resting and peak stress myocardial function and skeletal mass energetics [53]. Further clinical trials support a subjective improvement in symptoms of heart failure in patients treated with perhexiline [50,51,52]. The most recent available trial did produce contradictory evidence that there was no significant change in left ventricular function or BNP following perhexiline treatment, but did conclude that treatment improved cardiac energetics given the improvement in the phosphocreatine (PCr)/ATP ratio, a marker of heart failure, which correlates with the New York Heart Association (NYHA) class [50]. This novel evidence suggests that there may be a role of perhexiline in the metabolic modulation in heart failure management; now, the drug can be utilised safely, but a further larger scale study is required to confirm its use.

### 3.4. Etoximir

Etoximir is an inhibitor of CPT-1, similar to perhexiline. Initial animal studies of CPT-1 inhibition suggested that etoximir also improved cardiac function in diabetic rats, and hence, human clinical studies were performed [54]. Unfortunately, however, a randomized controlled trial etoximir vs. placebo of 347 patients had to be terminated early due unacceptably high liver transaminase levels in the etoximir group [55]. Following this study, due to safety concerns, etoximir was abandoned as a potential therapeutic agent in HF.

### 3.5. Malonyl CoA Decarboxylase Inhibition

MCD inhibitors increase myocardial malonyl coenzyme A levels. This leads to inhibition of CPT1, reducing fatty acid uptake and consequently increasing glucose metabolism similarly to perhexiline [56]. MCD inhibitors have been shown to have some beneficial effects in small studies in conditions, such as ischemia [57,58]; however, they have not yet been tested in the setting of heart failure.

### 3.6. Dichloroacetate

Dichloroacetate (DCA) inhibits pyruvate dehydrogenase kinase, increasing mitochondrial pyruvate dehydrogenase and thus increasing glucose oxidation and theoretically improving contractile function. There is some evidence to suggest that DCA may be associated with improved left ventricular function in animal studies [59]; however, older clinical trials in heart failure patients did not show any significant benefit, and the issue has not been studied further since [60,61].

## 4. Mitochondrial Dysfunction

Further to the metabolic disturbances contributing to the development and progression of heart failure, recent advances have emphasised the potentially key role of mitochondrial dysfunction in the pathophysiology of heart failure. This role is not yet well understood, and some contradictory evidence of the underlying mechanisms exists. However, it is likely that a combination of changes contribute to mitochondrial errors that ultimately lead to heart failure and that increased understanding and the adaptation of medications to target mitochondrial changes could be pivotal in advances in heart failure treatment.

A potential mechanism under review is the disruption of mitochondrial biogenesis, the production of new mitochondria, as an early event in the pathophysiology of heart failure [62]. Both human and rat experiments have determined a reduction in mitochondrial DNA (mtDNA) copy number and mitochondrial content in the failing myocardium [9]. Moreover, the early reversal of this disruption has been shown to be cardioprotective, suggesting a future target for therapy [62]. The generation of new mitochondria is complex and is regulated and stimulated by peroxisome proliferator-activated receptor gamma coactivator 1α (PCG1α), a nuclear-encoded protein, which is induced in states of increased energy demand to activate mitochondrial proliferation [62]. It is possible that the reduction in mitochondrial number observed in heart failure may be attributed to a diminution of PCG1α activity, but contradictory study results have been reported [62]. Further studies with a focus on cardiac hypertrophy have demonstrated that during early stages of compensated hypertrophy, mitochondrial biogenesis signaling is preserved in order to ensure increased energy supply given the increased cardiac workload [11]. In contrast, once decompensated heart failure becomes evident, a decline in mitochondrial biogenesis signals, including PCG1α, is observed, illustrating this change as contributory in the development of heart failure [11].

Overproduction of reactive oxygen species (ROS) has also been associated with the development of structural and functional changes occurring in myocardial failure [9]. The oxidative stress that arises in the setting of excess ROS has been causally linked to the progression of heart failure [63]. Many intracellular ROS, including superoxide and hydroxyl radicals and hydrogen peroxide (H_2_O_2_), are physiologically produced as a by-product of normal mitochondrial energy production. A variety of factors contribute to the excess production of ROS that occurs in heart failure. Oxidative stress is known to increase the activity of oxidase enzymes, which are partly responsible for increased levels of ROS [64]. A variety of studies have illustrated further contributory elements that lead to ROS accumulation in heart failure of different aetiologies, including hyperglycemia, reduction of antioxidants and oxidative injury following ischemia [65]. Moreover, once ROS excess develops, damage to cardiolipin, a key mitochondrial membrane phospholipid required to support mitochondrial energy production, occurs, stimulating a negative feedback with further ROS production and myocardial damage [14].

The role of ROS in the progression of heart failure is multifaceted and not fully understood. Mitochondria and mtDNA are most susceptible to ROS damage given that the majority of ROS is derived from the mitochondria and because the mitochondria lack histones, which are protective against ROS [9]. Consequently, with mitochondrial ROS damage, there is impaired mitochondrial functioning, including decreased transcription of mitochondrially-encoded subunits of the electron transport chain and a reduced capacity for oxidative phosphorylation [66]. This mitochondrial damage and dysfunction further aggravates the decreased energy metabolism observed in heart failure and potentiates the metabolic changes previously outlined [9]. Following ROS impairment of the mitochondria, cardiomyocyte function is inhibited in a myriad of ways, including in abnormalities of contractility, ion transport and calcium cycling [67]. Additionally, ROS are known to regulate signaling cascades, including protein kinase C (PKC), mitogen-activated protein kinase (MAPK), Jun N-terminal kinase and Ras, all of which are implicated in hypertrophy [9].

An alternate pathway that is also likely to be related to the mitochondrial influence on heart failure is changes in cardiolipin [14]. This role is closely related to that of ROS, as ROS are thought to fuel the oxidation of cardiolipin, resulting in reduced fatty acid oxidation and propensity for myocyte apoptosis [14]. Cardiolipin is important in the function of the ETC, as it binds cytochrome *c*, an electron carrier, to the inner mitochondrial membrane, permitting efficient electron transfer between complexes III and IV [68]. In the presence of ischemia, high calcium or high ROS, cardiolipin is peroxidised, negating its function in binding cytochrome *c* and reducing the mitochondrial energy production capacity [68]. A murine study has implicated a reduction of cardiolipin in the development of mitochondrial respiratory dysfunction and metabolic stress and noted that a selective loss of tetralinoleoyl cardiolipin, the predominant form in healthy mammalian hearts, occurs in heart failure that does not occur as part of the normal aging process in the absence of cardiac pathology [69].

## 5. Treatments Targeting Mitochondrial Dysfunction

In conjunction with metabolic modulation, recent efforts have focused on pursuing this increasingly understood mitochondrial disturbance in the production of pharmaceuticals that seek to rectify the underlying mechanisms. Further, it has also been revealed that the improved myocardial function that occurs with the currently available therapies may be partly attributable to the drugs’ effects on mitochondrial function [62]. For example, improved mitochondrial function has been recorded following treatment with ACE inhibitors and angiotensin receptor II blockers, medications proven to significantly improve survival when administered in heart failure [62]. Development of medications that specifically aim to influence the underlying mitochondrial deficits has also commenced.

### Elamipretide

Elamipretide, also referred to in the literature as Bendavia, Szeto–Schiller (SS)-31 or MTP-131, was one of the first drugs developed to selectively target the mitochondrial ETC to optimize energy efficiency [70]. Elamipretide is a Szeto–Schiller (SS) peptide; one of a family of compounds that was recently discovered by chance to selectively target the ETC to improve the efficiency of electron transport and restore cellular bioenergetics [70]. It is a water-soluble tetrapeptide composed of natural and synthetic amino acids, which is capable of crossing cellular membranes [71]. Elamipretide has a number of proposed mechanisms of actions. It is known to interact with the phospholipid cardiolipin, which is exclusively present within the inner mitochondrial membrane [72]. The likely mechanism is by the peptide binding to cardiolipin to prevent peroxidation by cytochrome *c* peroxidase, ensuring a preservation of mitochondrial cristae integrity [73]. In binding to cardiolipin, elamipretide also ensures close association of cardiolipin to cytochrome c, which is known to facilitate electron transport in the ETC and maximize energy production efficiency [74]. Further, animal studies have suggested that elamipretide restores the normal total levels of cardiolipin in the mitochondria when cardiolipin is depleted in the pathologic state [71]. Elamipretide also assists in diminishing intracellular ROS levels by reducing ROS production in the mitochondria in conjunction with acting to clear excess ROS [75]. For example, elamipretide treatment has been shown to reduce spontaneous generation of H_2_O_2_ in isolated mitochondria [76]. Elamipretide exerts its function on mitochondria across all organ systems and, so, has been investigated for use for a variety of diseases, including heart failure, ischemic heart disease, acute kidney injury and metabolic disorders [70].

**Figure 1:**
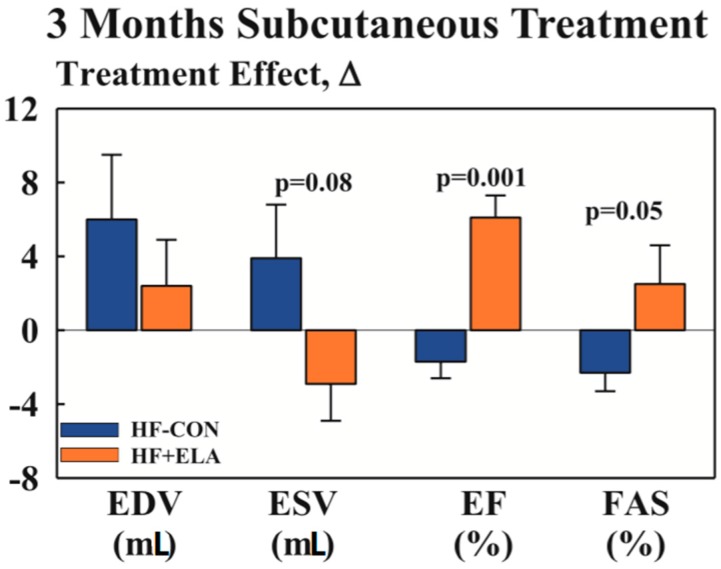
Change Δ (treatment effect) effect between pretreatment and 12 weeks post-treatment for left ventricular (LV) end-diastolic volume (EDV), end-systolic volume (ESV), ejection fraction (EF) and fractional area of shortening (FAS) in heart failure control dogs and heart failure dogs treated with elamipretide. Statistical significance based on *t*-statistics for two means. Bar graph depicted as mean ± SEM [71].

Despite a poverty of human clinical trials investigating the utilisation of elamipretide, a variety of animal studies has provided evidence supporting its use in heart failure, summarised in Table 5. In addition to outlining the mechanism of action of elamipretide, these animal studies have provided promising results that the drug may be clinically useful. A number of studies noted an improvement in left ventricular function in animals with induced heart failure treated with elamipretide [71,77]. Sabbah et al. demonstrated a significantly improved ejection fraction (Figure 1) in dogs with heart failure treated with elamipretide for a three month period [71]. It is also shown to prevent adverse left ventricular remodelling following ischemia, implicating a reduction in the development of heart failure post infarction [72,77]. Further, via restoration of cardiac myosin binding protein-C, elamipretide may contribute to improved left ventricular relaxation [78].

One human trial of use of elamipretide in humans is currently available. Daubert et al. aimed to ensure the safety of elamipretide treatment in patients with heart failure with reduced ejection fraction [79]. After administration of just a single dose, no subjects suffered any serious adverse events, and only one patient had to discontinue treatment due to non-serious adverse effects [79]. Further, the hemodynamic parameters of blood pressure and heart remained stable in all patients, suggesting that elamipretide may be tolerated in combination with current standard heart failure medications [79]. This trial, despite its small sample size, also resulted in a significant reduction in left ventricular volumes in patients treated with elamipretide compared to placebo [79]. Further, larger studies are required to fully investigate elamipretide to determine its safety in sequential dosing and its overall efficacy in heart failure, but it may be a useful therapeutic option for targeting mitochondrial dysfunction in the future. We are currently aware of one further randomised trial currently underway investigating the effect of multiple injections of elamipretide for patients with heart failure with reduced ejection fraction [81].

## 6. Conclusions

Improved understanding of the detailed pathophysiology of heart failure has prompted investigations for future therapeutics. Heart failure is recognised as a state of myocyte energy starvation due to metabolic changes that inhibit optimal energy production [8,13]. Medications including trimetazidine, ranolazine and perhexiline are aimed at rectifying the metabolic abnormalities in the failing heart, and the results of initial trials have been promising in indicating their role in heart failure management. Furthermore, there is also improved knowledge around the pivotal role of mitochondrial dysfunction in contributing to this energy deprivation. Utilisation of this knowledge has only recently commenced, but elamipretide is likely to be the first of many medications targeting this area given the exciting improvements to left ventricular function it has made in initial investigations. Greater evidence, in the form of large randomised controlled trials, is required to confirm the role of elamipretide and metabolic-modulating drugs in the treatment of heart failure, but it is expected to be an area of great advances in the future.

## Figures and Tables

**Table 1:** Summary of recent trials and previous meta-analyses investigating the use of trimetazidine in heart failure patients.

Study	Year	Study Design	No. of Patients	Patient Cohort	Results
Li, Li [28]	2016	RCT with follow up period of 12 weeks	140	Patients with coronary heart disease and heart failure	Treatment with trimetazidine and metoprolol in addition to conventional treatment compared to standard treatment alone resulted in: Greater improvement in BNP (t = 19.41 pg/mL, <0.01) Improved LVEF (t = 1.683%, *p* < 0.05)
Grajek, Michalak [29]	2015	Meta-analysis of 3 RCTs	326	Patients with heart failure of various aetiologies and stages	Treatment with trimetazidine compared to placebo resulted in a reduction of all-cause mortality (RR 0.283, *p* < 0.0001)
Zhou, Chen [30]	2014	Meta-analysis of 19 RCTs	1042	Patients with heart failure of various aetiologies and stages	Treatment with trimetazidine compared to conventional treatment alone resulted in: LVEF improvement (WMD 7.29%, *p* < 0.01) Improved NYHA classification (WMD −0.55, 95% CI −0.81–−0.28; *p* < 0.01) No difference in exercise tolerance (WMD 18.58, *p* = 0.15) No difference in all-cause mortality (RR 0.47; 95% CI 0.12–1.78; *p* = 0.27) Reduction in BNP (WMD −157.08 pg/mL; CI −176.55–−137.62; *p* < 0.01)
Zhang et al. [22]	2012	Meta-analysis of 16 RCTs	884	Patients with heart failure of various aetiologies and stages	Treatment with trimetazidine compared to placebo resulted in: No difference in all-cause mortality (RR 0.47, *p* = 0.27) Improved LVEF (WMD 6.46%, *p* < 0.0001) Reduced NYHA functional class (WMD −0.57, *p* = 0.0003) Improved exercise tolerance (WMD 63.75 s, *p* < 0.0001) Downregulation of BNP (WMD −203.4 pg/mL, *p* = 0.0002)
Fragasso et al. [31]	2012	Multi-centre retrospective cohort study	669	Patients with systolic-diastolic heart failure with EF < 45% and NYHA Class II–IV	Treatment with trimetazidine compared to conventional treatment alone (after propensity score was performed) resulted in: Mortality risk reduction (HR 0.189; 95% CI 0.079–0.454; *p* = 0.0002) Reduction in CVD death (HR 0.072; 95% CI 0.019–0.286; *p* = 0.0001) 10.4% reduction in hospitalization (*p* < 0.0005)
Gao et al. [32]	2011	Meta-analysis of 17 randomised studies	955	Patients with heart failure of various aetiologies and stages	Treatment with trimetazidine compared to placebo resulted in: Improved LVEF (WMD 7.49%, *p* < 0.01) Reduced NHYA classification (WMD −0.41, *p* < 0.01) Increased exercise tolerance (WMD 30.26 s, *p* < 0.01) Reduced mortality (RR 0.29; 95% CI 0.17–0.49; *p* < 0.01) Reduced cardiovascular events and hospitalisations (RR 0.42; 95% CI 0.30–0.58; *p* < 0.01)

RCTs = randomised control trials; BNP = brain natriuretic peptide; LVEF = left ventricular ejection fraction; RR = relative risk; WMD = weighted mean difference; NHYA = New York Heart Association; CVD = cardiovascular.

**Table 2:** Summary of clinical trials investigating the potential use of ranolazine in heart failure.

Study	Year	Study Design	No. of Patients	Patient Cohort	Results
Murray, Colombo [36]	2014	Unblinded, non-randomised trial	109	Systolic or diastolic heart failure patients with NYHA Class II–IV	Treatment with ranolazine compared to standard heart failure therapy alone resulted in: Increased LVEF (>7 EFU, *p* < 0.001) Cardiovascular event rate reduction
Maier et al. [34]	2013	Prospective, randomised, double-blind, placebo-controlled proof-of-concept study	20	Patients with diastolic heart failure with preserved ejection fraction (EF > 45%)	In comparison to placebo, treatment with ranolazine in heart failure resulted in: Reduced left ventricular end-diastolic pressure (2.2 mmHg, *p* = 0.04) Reduction in cardiac out (0.3 l/min) and stroke volume (3.3 mL) (*p* = 0.04) No difference in exercise tolerance No difference in BNP levels
Morrow et al. [33]	2010	Randomised, double-blind, placebo-controlled trial	4543	Non-ST-segment elevation ACS patients	Treatment with ranolazine compared to placebo resulted in: 13% reduction in the rate of recurrent ischemia (HR 0.87; 95% CI 0.76–0.99; *p* = 0.03) No difference in incidence of new or worsening heart failure No difference in exercise performance No change in BNP concentration

NYHA = Ney York Heart Association; LVEF = left ventricular ejection fraction; EFU = ejection fraction units; BNP = brain natriuretic peptide; ACS = acute coronary syndrome.

**Table 3:** Non-clinical experiments examining the benefits of ranolazine in heart failure.

Study	Year	Study Design	Results
Sossalla et al. [39]	2008	Treatment of myocytes of 10 isolated failing human hearts with ranolazine	Treatment with ranolazine resulted in a reduction of the diastolic tension (3.9 mN/mm^2^ reduction, *p* < 0.05)
Rastogi et al. [42]	2008	Canine study of 28 dogs with induced heart failure with a randomised, blinded, placebo-controlled design	Treatment with ranolazine alone in heart failure resulted compared to placebo in: Improved EF by 2% (*p* ≤ 0.05) Reduction of EDWS by 14 gm/cm^2^ Treatment with combination of ranolazine and enalapril compared to placebo resulted in: Improved EF by 5% (*p* ≤ 0.05) Reduction of EDWS by 13 gm/cm^2^ Treatment with combination of ranolazine and metoprolol compared to placebo resulted in: Improved EF by 7% (*p* ≤ 0.05) Reduction of EDWS by 20 gm/cm^2^
Undrovinas et al. [40]	2006	Treatment of 26 isolated canine hearts post induction of heart failure	Ranolazine treatment of isolated canine hearts with induced heart failure resulted in: Restoration of normal myocyte relaxation Reduction in the resting tension of the myocytes
Sabbah et al. [41]	2002	Canine study of 21 dogs with induced heart failure	Treatment of dogs with heart failure with ranolazine resulted in: Reduction of LVEDP by 3 mmHg (*p* = 0.001) Increased cardiac output of 0.39 L/min (*p* = 0.01)

EF = ejection fraction; EDWS = end-diastolic circumferential wall stress; LVEDP = left ventricular end-diastolic pressure.

**Table 4:** Summary of the clinical trials investigating the use of perhexiline in the management of heart failure.

Study	Year	Study Design	No. of Patients	Patient Cohort	Results
Beadle et al. [50]	2015	Randomised double-blind placebo-controlled trial, parallel-group study	47	Patients with systolic heart failure of non-ischemic etiology with NYHA class of II–IV	Treatment of with perhexiline compared to placebo in heart failure resulted in: 30% increase in PCr/ATP ratio (*p* < 0.001) (no change in placebo group) 52% of treated patients improved by 1 NHYA class compared to 20% improving in the placebo group (*p* = 0.02) No change in LVEF (*p* = 0.68) No change in BNP levels
Abozguia et al. [51]	2010	Randomised, double-blind, placebo-controlled, parallel-group trial	46	Patients with symptomatic exercise limitation caused by non-obstructive hypertrophic cardiomyopathy	Treatment of patients with hypertrophic cardiomyopathy with perhexiline compared to placebo resulted in: Improved exercise capacity (VO_2_ increased by 2.1 mL/kg/min, *p* = 0.003) Reportedly less symptoms (MLHFQ score improved by 8, *p* < 0.001) Improved NYHA classification in more patients (67% of treated patients compared to 30% of control) No significant difference in ejection fraction
Phan et al. [52]	2009		151	Patients with chronic heart failure (LVEF < 40% with NYHA class > IIb) or refractory angina	Treatment of patients with angina or heart failure with perhexiline resulted in the majority of patients reporting subjective symptom reduction (58.9%)
Lee et al. [53]	2005	Randomised double-blind placebo-controlled trial	56	Patient with chronic heart failure with EF < 40% and NYHA Class II or III already on optimal treatment	Treatment of with perhexiline compared to placebo in heart failure resulted in: Increased VO_2_ max by 17% (*p* < 0.001) (no change in placebo group) Increased LVEF by 10% (*p* < 0.001) with no change in the placebo group Reduced symptoms of heart failure (MLHFQ score reduced by 24%, *p* = 0.04) while placebo group score was unchanged Mean NYHA classification improved by 21% (*p* = 0.02) with no change in the placebo group

PCr/ATP = phosphocreatine/adenosine triphosphate; NHYA = New York Heart Association; LVEF = left ventricular ejection fraction; BNP = brain natriuretic peptide; VO_2_ max = peak exercise oxygen consumption; MLHFQ = Minnesota Living with Heart Failure Questionnaire.

**Table 5:** Experimental trials investigating the role of elamipretide in heart failure treatment.

Study	Year	Study Design	No. of Patients	Results
Daubert et al. [79]	2016	Phase I randomised, placebo-controlled trial	36 patients with stable heart failure (EF < 45% and NYHA Class II–III)	Heart failure treated with elamipretide compared to placebo resulted in: Reduced left ventricular end-systolic volume (between-group difference −13.7; 95% CI −22.7–−4.8; *p* = 0.005) and end-diastolic volume (between-group difference −17.9 mL; 95% CI −30.6–−5.2; *p* = 0.009) Elamipretide was well tolerated with no influence on blood and pressure and heart rate
Sabbah et al. [71]	2016	Canine experiment	14 dogs	Treatment of dogs with heart failure with elamipretide compared to intravenous saline resulted in: Improved EF (6% increase compared to pretreatment, *p* < 0.05) with no change in control Reduced end-systolic LV volume (3 mL, *p* < 0.05) compared to increased volume in the control group Increased maximum rate of ATP synthesis and increased ATP/ADP ratio (*p* < 0.05) No effect on heart rate, mean aortic pressure, systemic vascular resistance or LV end-diastolic volume
Gupta et al. [78]	2016	Canine experiment	14 dogs	Treatment of dogs with heart failure with elamipretide resulted in restoration of near normal levels of cMyBPC-S282 in the left ventricle (*p* < 0.05)
Shi et al. [72]	2015	Murine experiment	24 rats	Treatment of post-MI rats with elamipretide showed: Restored gene expression of mitochondrial energy metabolism Promotion of mitochondrial biogenesis Regulation of glucose and fatty acid oxidation related gene expression Preserved SERCA2a expression Reduction in cardiac fibrosis
Dai et al. [77]	2014	Murine experiment	56 rats	Rats treated with elamipretide compared to water after acute MI showed improved LV function and prevention of adverse left ventricle remodelling.
Sabbah et al. [80]	2014	Canine experiment	12 dogs	Dogs with heart failure treated with elamipretide compared to normal saline resulted in normalised expression of cardiolipin-remodelling genes and proteins (*p* < 0.05)

EF = ejection fraction; LV = left ventricle; cMyBPC-S282 = cardiac myosin binding protein-C at serine 282; SERCA2a = sarco/endoplasmic reticulum; MI = myocardial infarct.
